# Production of Thermostable Xylanase by *Myceliophthora heterothallica* in Solid-State Culture Using Agro-Industrial Residues

**DOI:** 10.3390/jof12070461

**Published:** 2026-06-24

**Authors:** Eduardo da Silva Martins, Dreison Mendanha Leal Arouca Poço, Heytor Lemos Martins

**Affiliations:** 1 Microbiology Laboratory, Department of Agricultural and Biological Sciences, State University of Minas Gerais, Frutal CEP 38202-436, MG, Brazil; eduardo.martins@uemg.br (E.d.S.M.); dreisonarouca@hotmail.com (D.M.L.A.P.); 2 Department of Biology, School of Agricultural and Veterinary Sciences, São Paulo State University (UNESP), Jaboticabal 14884-900, SP, Brazil

**Keywords:** *Thermophilic fungus*, enzyme, xylanolytic activity

## Abstract

Xylanases are enzymes used in the conversion of lignocellulosic substances to fuels, digestion of animal feed, food and textile industries and as bleaching agents in paper production. The present study evaluated the production of a thermostable xylanase of the thermophilic fungus *Myceliophthora heterothallica* by solid-state fermentation of agro-industrial residues (sugarcane bagasse, sugarcane straw, wheat bran, and a mixture of the three substrates (1:1:1 *w*/*w*). Different cultivation parameters for the production of the enzyme were evaluated. The highest production of xylanase occurred in the mixture of the three substrates, after 4 days of cultivation. The activity of the enzyme was higher in the following conditions: water at pH 5.0 and incubation temperature of the fungus at 40 °C, with initial substrate moisture at 80%. The enzyme presented higher activity in the pH range between 5.0 and 6.5, with a peak at pH 5.0 and over 90% stability over a wide pH range (3.5 to 9.5). The optimum temperature was 65 °C and the enzyme showed 100% stability for 1 h, up to 60 °C. The results demonstrate that agro-industrial residues are efficient substrates for xylanase production, allowing the production of an enzyme with high stability under pH and temperature variations, a feature essential for its application in industrial processes.

## 1. Introduction

The use of agro-industrial waste and renewable raw materials from biomass from various sources is of fundamental importance for the construction of various sectors of the bioeconomy, based on innovations in the areas of food, biofuels, health and the environment, in which different bioproducts can be used. Worldwide, the use of residual biomass has been increasingly explored, since the development of biotechnological processes allows adding value to these materials [1].

Solid-state cultivation has shown promise in the development of various bioprocesses, such as bioremediation and biodegradation of toxic compounds; detoxification of agro-industrial residues; and biotransformation of crop residues for nutritional enrichment and in obtaining high-value-added products, such as secondary metabolites (antibiotics, alkaloids, plant growth factors, etc.), organic acids, biopesticides, biofuels, aromatic compounds and enzymes [2].

Xylanases (EC3.2.1.8) are hydrolytic enzymes that cleave the β-1,4 bonds of the polysaccharide xylan, the main component of hemicellulose and one of the main constituents of the plant cell wall [3]. Together with cellulases and pectinases, xylanases are responsible for about 20% of the world market for enzymes, since they have a diversified industrial application, including the production of food, medicines, sweeteners, solvents, fuels and even the bleaching of paper pulp [4,5]. The development and improvement in technologies aimed at the production of xylanases with biochemical characteristics suitable for industrial application have been the objective of many studies. In addition, there is a growing need for product and process optimization; minimizing costs and time; and maximizing yield, productivity, and quality of bioproducts [6].

One of the factors that most affect the cost of producing enzymes is the substrate. Thus, the use of agro-industrial residues in the cultivation of microorganisms to obtain the enzyme, in addition to being economically viable, can help solve environmental problems arising from their accumulation in nature [7,8,9]. In this context, the use of agro-industrial waste becomes attractive, because in addition to preventing possible problems related to its accumulation and inadequate management, it can reduce the costs of enzymatic production, since they are usually generated in large quantities. In addition to the type of substrate, several factors can influence the production of fungal enzymes, such as incubation time, pH, moisture content, and temperature, which must be optimized for higher productivity [10].

In recent years, thermophilic fungi have attracted special attention, since the enzymes produced by these organisms can present greater thermostability, allowing the saccharification of polysaccharide biomass at higher temperatures and reducing the reaction time and viscosity of the substrate [11]. *Myceliophthora* is a genus composed of mesophilic and thermophilic fungi, which includes 10 species. *M. heterothallica* has been described as thermophilic and considered a producer of enzymes of industrial interest due to its high activity rates and thermostability [12].

Recent studies have demonstrated increasing interest in xylanase production through solid-state fermentation using agro-industrial residues. Wheat bran, sugarcane bagasse, sugarcane straw and other lignocellulosic materials have been widely explored as low-cost substrates for enzyme production. Rodrigues [13] reported xylanase production by *Bacillus* sp. cultivated on wheat bran under solid-state fermentation, while [14] described the production of a thermotolerant xylanase from *Rasamsonia composticola* using agro-residues and demonstrated its application in sugarcane bagasse saccharification. Nunes [15] reported efficient xylanase production by *Penicillium* sp. FSDE15 using agro-industrial residues, whereas Abena and Simachew [16] characterized an acidophilic xylanase produced by a white-rot fungus cultivated on wheat straw. More recently, Soltero-Sánchez et al. [17] demonstrated the potential of water hyacinth and sugarcane bagasse as substrates for the enhanced production of xylanases and cellulases under solid-state fermentation. Additionally, agro-industrial and food-processing residues have been recognized as promising feedstocks for the sustainable production of hydrolytic enzymes through solid-state fermentation processes [18].

Among thermophilic fungi, species of the genus *Myceliophthora* have attracted considerable attention due to their ability to produce thermostable lignocellulolytic enzymes. Simões [19] purified and characterized a thermostable xylanase secreted by *Myceliophthora heterothallica* F.2.1.4, reporting favorable biochemical properties for industrial applications. More recently, De Amo [20] successfully expressed a GH11 xylanase from *M. heterothallica* in *Pichia pastoris*, highlighting the growing interest in the biotechnological potential of xylanases produced by this species. Despite these advances, information regarding xylanase production by *M. heterothallica* using agro-industrial residues under solid-state fermentation remains limited, particularly concerning the optimization of cultivation parameters and cultivation conditions affecting enzyme yield and stability.

To the best of our knowledge, there are no reports describing the optimization of xylanase production by *Myceliophthora heterothallica* using a combination of sugarcane bagasse, sugarcane straw and wheat bran under solid-state fermentation conditions. Furthermore, information regarding the influence of cultivation parameters on enzyme production and the biochemical properties of the crude xylanase produced from these agro-industrial residues remains scarce. Therefore, this study provides new insights into the use of abundant sugarcane-derived residues for the production of thermostable xylanases with potential industrial applications.

In view of the above, this work aimed to evaluate the use of agro-industrial residues and the effect of different cultivation conditions on xylanase production by the thermophilic fungus *Myceliophthora heterothallica* and to characterize the enzyme.

## 2. Materials and Methods

### 2.1. Microorganism

The thermophilic fungus *Myceliophthora heterothallica*, from the Laboratory of Biochemistry and Applied Microbiology of UNESP, São José do Rio Preto campus, was studied. For periodic rebounds and conservation of pure cultures, the nutrient medium Sabouraud with ampicillin was used. Pure cultures were kept in cryovials, under 20% glycerol solution, in a freezer at −80 °C.

### 2.2. Enzyme Production by Solid-State Culture (CES)

For each culture by CES, one pre-inoculum was prepared in a 250 mL Erlenmeyer flask containing 100 mL of Sabouraud agar. The fungus was inoculated on the surface of this medium by striations, and incubated at 45 °C until complete growth (mycelial or sporulation).

The cultures were conducted in Erlenmeyer flasks (250 mL), using 5 g of the following substrates: sugarcane bagasse (BC), sugarcane straw (PC), wheat bran (FT) and a mixture of these three materials (1:1:1 *w*/*w*) (called MIX). The sugarcane bagasse and straw were acquired from mills in the municipality of Frutal/MG. The wheat bran of local commerce. The substrates were washed, dried at 60 °C in a drying oven and sieved at 10 mesh.

The inoculum was standardized with the addition of 4 mycelial disks (1 mycelial disk = 0.5 cm^2^) for each vial containing 5 g of substrate. Initially, the nutrient solution used for supplementation of the substrates was composed of NH_4_NO_3_, MgSO_4_·7H_2_O and (NH_4_)_2_SO_4_ (0.1%), pH 5.0, so that the initial moisture of the substrate was 75%. The fungus was cultured at 45 °C. Every 24 h, samples were taken, up to 240 h (10 days).

The agro-industrial residues employed in this study present physicochemical characteristics favorable for fungal growth and xylanase production (Table 1). Sugarcane bagasse contains approximately 32–45% cellulose, 20–32% hemicellulose and 17–32% lignin, whereas sugarcane straw contains approximately 33–40% cellulose, 25–28% hemicellulose and about 21% lignin. Wheat bran is particularly rich in hemicellulosic compounds, containing 41–60% non-starch polysaccharides, in addition to proteins, minerals and other nutrients that support microbial growth and enzyme synthesis. The elevated hemicellulose content of these materials makes them suitable substrates for xylanase production under solid-state fermentation conditions.

To each sample taken, 80 mL of distilled water was added, and the mixture was manually homogenized and kept under shaker agitation (100 rpm) for 20 min. After this period, the material was filtered in a nylon tissue disk, centrifuged at 5000× *g* for 15 min at 5 °C, and the supernatant was used to determine the enzymatic activities.

#### Cultivation Parameters

To evaluate the effect of substrate supplementation (chosen in the previous step) on enzyme production, the following nutrient solutions were used next to the substrate: 1—0.1% NH_4_NO_3_; 2—(NH_4_)_2_SO_4_ at 0.1%; 3—NH_4_NO_3_, MgSO_4_·7H_2_O, (NH_4_)_2_SO_4_ (all at 0.1%); 4—0.1% yeast extract; and 5—water (control), so that the initial humidity was 75%. The pH of the solutions was adjusted to 5.0.

The pH values of each solution were evaluated from 4.0 to 6.0 (with a variation of 0.5 in 0.5), in a 5 × 5 factorial design.

To evaluate the effect of the initial moisture of the substrate, volumes of nutrient solution were added to the inoculum, so that the initial moisture was 60%, 65%, 70%, 75% and 80%. The fermentation temperatures evaluated were 40 °C, 45 °C, 50 °C and 55 °C, in 5 × 4 factorial design.

### 2.3. Biochemical Characterization of Xylanase

#### 2.3.1. Effect of pH and Temperature on Enzyme Activity

To determine the optimal pH, xylanase activity was evaluated by incubating 0.1 mL of the enzymatic solution in 0.9 mL of 1% Beechwood xylan suspension (Sigma^®^ (Kanagawa, Japan)—Merk Brazil (São Paulo, Brazil)—Sigma Aldrich (St. Louis, MO, USA)) at different pH values at 60 °C for 10 min. The following buffers (0.1 M) were used: sodium citra characterization enzyme (pH 3.0), sodium acetate (pH 3.5–5.5), MES (pH 6.0–6.5), HEPES (pH 7.0–7.5), glycine-NaOH (pH 8.0–9.5) and CAPS (pH 10.0–10.5).

The effect of temperature on enzymatic activity was evaluated by varying the temperature between 40 and 80 °C, with variations of 5 °C. The activity assays were performed at the optimal pH verified for the enzyme. The methodology was adapted from [19].

#### 2.3.2. Effect of pH and Temperature on Enzyme Stability

To determine the stability of the enzyme, a final volume of 1.0 mL of the enzyme extract was incubated at 25 °C for 24 h. After the incubation period, the enzymatic activity was measured at the optimal temperature and pH of each enzyme.

Thermostability was evaluated by incubating the enzyme for one hour at temperatures from 10 to 80 °C, followed by determination of residual activity, according to the optimal pH and temperature conditions. The methodology was adapted from [19].

### 2.4. Determination of Xylanase Activity

The xylanase activity was determined in a reaction mixture containing 0.1 mL of enzymatic solution and 0.9 mL of Beechwood xylan substrate solution (Sigma^®^, Kanagawa, Japan) (10.0 g L^−1^) prepared in 0.10 M acetate buffer (pH 5.0). The reaction mixture was incubated at 60 °C for 10 min and then stopped by the addition of 1.0 mL of DNS reagent (3,5-dinitrosalicylic acid). Reducing sugars released during xylan hydrolysis were quantified according to the method proposed by Miller (1959) [24], using a standard xylose calibration curve.

One unit of xylanase activity (U) was defined as the amount of enzyme required to release 1.0 μmol of reducing sugar per minute under the assay conditions described above. Xylanase activity was expressed as units per gram of dry substrate (U·g^−1^ dry substrate), corresponding to the total enzymatic activity recovered from the crude extract divided by the initial dry mass of substrate used in the solid-state fermentation (5 g).

Xylanase activity was calculated according to Equation (1):

Activity (U·g^−1^) = (C × V)/(t × m) (1)

where C is the amount of sugars released (μmol), V is the total volume of crude enzyme extract (mL), t is the reaction time (min), and m is the initial dry mass of substrate (g).

### 2.5. Statistical Analysis of the Data

With the data obtained from the different treatments, analysis of variance (ANOVA) was performed and then the Scott–Knott test was applied at 5% probability to compare the means.

## 3. Results and Discussion

### 3.1. Xylanase Production by M. heterothallica in Different Substrates and Cultivation Conditions

**Figure 1:**
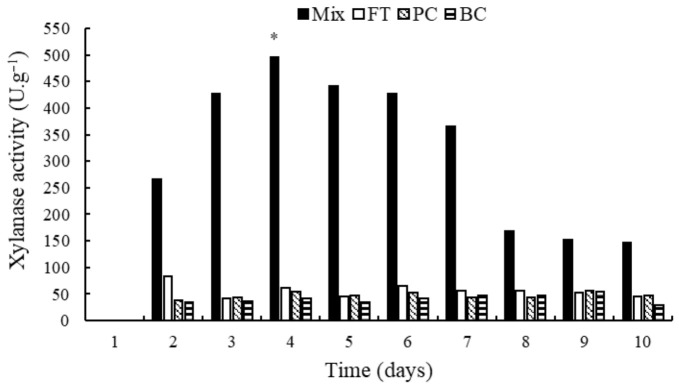
Xylanase production by *Myceliophthora heterothallica* in different substrates and cultivation times. FT: wheat bran; PC: sugarcane straw; BC: sugarcane bagasse; MIX: mixture of the three substrates (1:1:1 *w*/*w*). * Condition in which there was greater enzymatic activity, with a statistical difference in the Scott–Knott test (<0.05) in relation to the other treatments.

The highest xylanase activity occurred in the medium containing the mixture of sugarcane bagasse, sugarcane straw and wheat bran (MIX), after 4 days of cultivation (495.2 U·g^−1^), a condition that showed a statistical difference in relation to the other values presented. When the fungus was inoculated in the other media in isolation, the xylanase activity values were significantly lower (Figure 1), suggesting that components of these substrates complement each other, favoring the production of xylanase by the fungus.

The highest enzymatic activity was recorded when the combination of wheat bran, sugarcane bagasse and sugarcane straw was used as substrate, possibly due to the complementarity between the physicochemical characteristics of these materials. Wheat bran is widely used in the production of xylanases, mainly because it has a high content of hemicellulose, especially arabinoxylans, in addition to having a good moisture retention capacity, a determining factor for microbial growth in solid-state cultivation (CES) processes [25,26].

On the other hand, sugarcane bagasse, although it presents greater resistance to degradation due to its complex lignocellulosic matrix, contributes significantly to the improvement of the physical properties of the medium. Its fibrous structure favors aeration, allowing better gas exchange and dissipation of heat generated during microbial cultivation [27]. Thus, the joint use of these residues provided adequate conditions for the development of the fungus and xylanase expression. Sugarcane straw also contributed to enzyme production by supplying additional hemicellulosic fractions and improving moisture retention and substrate porosity. The presence of straw may have increased the structural heterogeneity of the cultivation matrix, favoring oxygen diffusion and fungal colonization throughout the substrate.

The mixture provides a balance between the availability of nutrients, such as xylans present in wheat bran, and desirable physical characteristics, such as the porosity conferred by sugarcane bagasse, resulting in greater enzymatic activity [28]. Previous studies have shown that the use of combined substrates often results in higher enzymatic yields when compared to the use of isolated materials, due to the complementarity of carbon and nitrogen sources, the presence of micronutrients and the physical structure of the growing medium [29].

Knob et al. [30] reported that wheat bran is one of the most frequently used substrates for fungal xylanase production under solid-state fermentation. Its high content of hemicellulosic compounds, particularly arabinoxylans, provides important inducers for xylanase synthesis while also supporting fungal growth. In addition, the authors highlighted solid-state fermentation as an attractive strategy for the valorization of agro-industrial residues, including sugarcane bagasse, contributing to both enzyme production and waste management.

**Figure 2:**
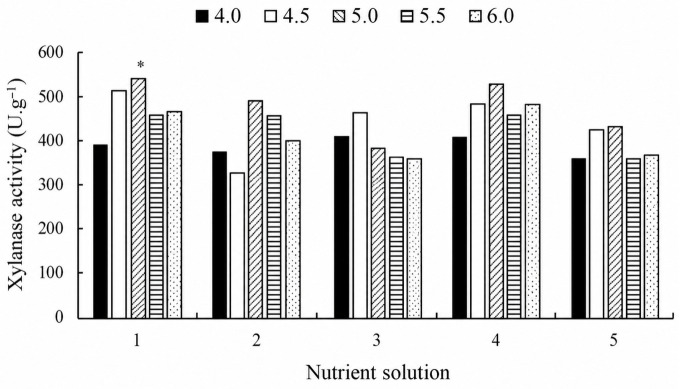
Xylanase production by *Myceliophthora heterothallica* in a substrate composed of wheat bran, sugarcane bagasse and sugarcane straw (1:1:1 *w*/*w*), supplemented with different nutrient solutions at different pH values, in 4 days of cultivation. 1—water; 2—NH_4_NO_3_ at 0.1%; 3—(NH_4_)_2_SO_4_ at 0.1%; 4—NH_4_NO_3_, MgSO_4_·7H_2_O, (NH_4_)_2_SO_4_ (all at 0.1%); 5—yeast extract at 0.1%; 5—water (control). * Condition in which there was a statistical difference in the Scott–Knott test (<0.05) in relation to the other treatments.

When different nutrient solutions supplementary to the substrate were evaluated at different pH values, it was observed that the highest xylanase activity (533.3 U·g^−1^) occurred when the substrate was supplemented only with distilled water, at pH 5.0, with a statistically significant difference for the other conditions, as shown in Figure 2.

The supplementation of the substrates with distilled water was sufficient to sustain the production of xylanase, with emphasis on pH 5.0, which presented the highest enzymatic activity. This result indicates that the combination of wheat bran, sugarcane bagasse and sugarcane straw provides adequate nutrients and physical support for the development of the microorganism, even in the absence of complex nutrient solutions.

Wheat bran has proteins, minerals and vitamins, in addition to a high hemicellulose content, which characterizes it as an efficient substrate to produce hemicellulolytic enzymes [25,26]. Sugarcane bagasse, although more lignified, contributes to the aeration of the medium, favoring the stability of the fermentation process. The absence of a significant increase with the addition of nutrient solution may be related to the saturation of the nutritional requirements of the microorganism [31].

Regarding the initial pH of the substrate, some studies report the preference of fungi for substrates with a slightly acidic initial pH in the production of xylanases, such as in the enzymes produced by *Aspergillus niger* [32] and *white rot fungi* [16].

**Figure 3:**
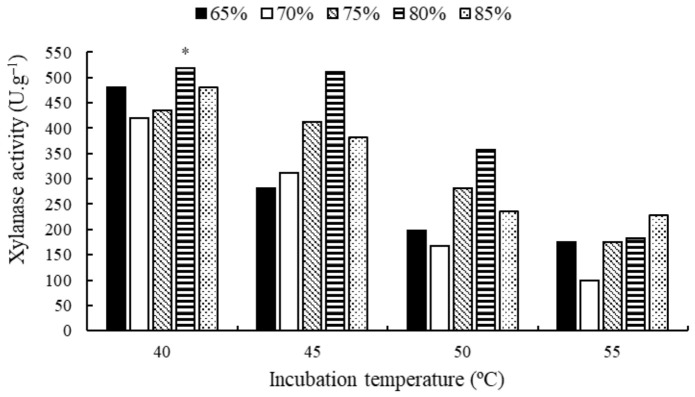
Xylanase production by *Myceliophthora heterothallica* in a substrate composed of wheat bran, sugarcane bagasse and sugarcane straw (1:1:1 *w*/*w*), hydrated with sterilized distilled water, pH 5.0, in 4 days of cultivation, under different conditions of humidity and incubation temperature of the fungus. * Condition in which there was a statistical difference in the Scott–Knott test (<0.05) in relation to the other treatments.

Under the best conditions obtained, an experiment was carried out to evaluate the effect of the initial moisture of the substrate and the incubation temperature of the fungus. It was observed that the highest xylanase activities occurred with the fungus incubated at 40 °C and with initial substrate moisture of 80% (Figure 3).

The higher enzymatic activity observed in the condition of 80% moisture indicates that the substrate used has a high-water retention capacity, in addition to favoring the growth of the evaluated fungus, which demonstrates adaptation to more humid environments. This trait is often reported for thermophilic fungi employed in solid-state culture (CES) [31]. In addition, the combination of wheat bran, sugarcane bagasse and sugarcane straw may have contributed to the maintenance of an adequate physical structure of the medium, even under high humidity, avoiding substrate compaction and allowing adequate aeration, an essential factor for fungal metabolism in CES.

The best performance observed at 40 °C is in line with the physiological profile of thermophilic microorganisms and with previously described results to produce xylanases. High temperatures, as long as they are within the tolerance range of the microorganism, tend to favor both the enzymatic activity and the gene expression of extracellular enzymes [33]. On the other hand, temperatures above the optimum can compromise the stability of the enzymes produced or inhibit cell growth, which explains the reduction in enzymatic activity observed in the higher temperature ranges.

The maximum xylanase activity obtained under the optimized cultivation conditions (533.3 U·g^−1^ dry substrate) demonstrates the potential of *Myceliophthora heterothallica* for enzyme production using agro-industrial residues under solid-state fermentation. This activity was considerably higher than that reported for *Penicillium* sp. FSDE15 cultivated on wheat bran, which reached 102.34 U·g^−1^ under optimized conditions [15]. However, higher activities have been reported for *Aspergillus austwickii* B6 (752 U·g^−1^ dry matter), *Trichoderma harzianum* M7 (1724 U·g^−1^ dry matter) [17], and *Bacillus* sp. TC-DT13 (2943 U·g^−1^) cultivated on wheat bran [13]. Differences among studies are expected because xylanase production is strongly influenced by microbial strain, substrate composition, cultivation conditions and extraction procedures.

In the present study, the substrate mixture composed of sugarcane bagasse, sugarcane straw and wheat bran likely promoted a synergistic effect by combining structural lignocellulosic components with nutrient-rich materials. While sugarcane residues provide cellulose and hemicellulose fractions capable of inducing xylanase synthesis, wheat bran supplies proteins, vitamins, minerals and readily available carbohydrates that support fungal growth and metabolism. Furthermore, the thermophilic nature of *M. heterothallica* and the known thermostability of its xylanases represent important advantages for industrial applications, reinforcing the biotechnological potential of this microorganism.

In addition to substrate composition, the physiological characteristics of *M. heterothallica* may have contributed to the observed enzyme production. This fungus is classified as thermophilic and has been recognized as a promising source of lignocellulolytic enzymes with industrial relevance. Thermophilic fungi frequently exhibit enhanced metabolic activity at elevated temperatures, reducing contamination risks during cultivation and improving process robustness. Previous studies demonstrated that xylanases produced by *M. heterothallica* exhibit favorable biochemical properties, including broad pH tolerance and high thermal stability [19,20], characteristics that are highly desirable for industrial biomass conversion processes.

From an industrial perspective, the production of xylanase using sugarcane bagasse, sugarcane straw and wheat bran represents an attractive strategy for the valorization of agro-industrial residues. Brazil is one of the world’s largest producers of sugarcane, generating substantial quantities of lignocellulosic by-products annually. The conversion of these materials into high-value bioproducts such as industrial enzymes contributes to waste reduction, process sustainability and the development of biorefinery concepts based on circular economy principles. Therefore, the optimized production conditions established in this study provide a promising basis for future scale-up studies and industrial applications of *M. heterothallica* xylanase.

### 3.2. Biochemical Characterization of M. heterothallica Xylanase

**Figure 4:**
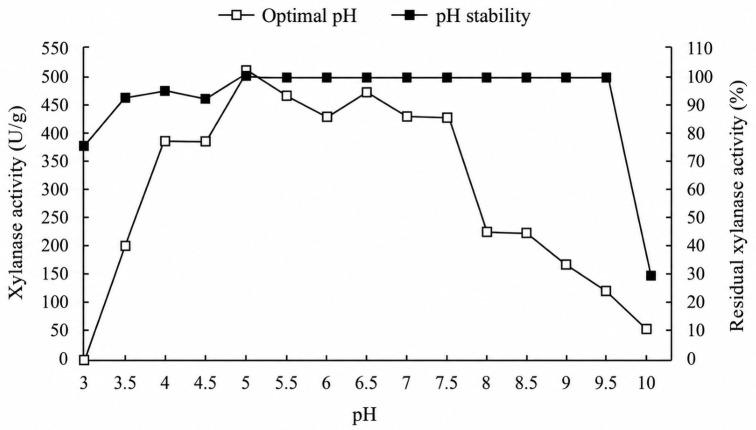
Effect of pH on the xylanase activity of the fungus *Myceliophthora heterothallica*. -□- optimal pH; -■- pH stability for 24 h at 8 °C.

The xylanase of *M. heterothallica* showed the highest activity in the pH range between 4.0 and 7.5, with a peak at pH 5.0. The enzyme showed high stability at pH for 24 h, maintaining more than 90% of the activity between pH 3.5 and 4.5 and 100% of the activity at pH values between 5.0 and 9.5 (Figure 4). This is a desirable characteristic for an enzyme for industrial application, since it has activity and stability over a wide pH range.

The activity and stability profile of the xylanase produced by *Myceliophthora heterothallica* reveals desirable characteristics for industrial applications, especially those requiring variable pH operating conditions. In this study, the enzyme showed maximum activity at pH 5.0 and maintained high levels of activity (above 90%) in a wide pH range between 4.0 and 7.5. In addition, it demonstrated high stability, maintaining more than 90% of the activity after 24 h at pH between 3.5 and 4.5, and 100% between pH 5.0 and 9.5.

Several studies have shown that xylanases produced by filamentous fungi have optimal activity in slightly acidic pH ranges, as observed in xylanases of *Aspergillus japonicus* [34], *Paecilomyces variotii* [35] and *Aspergillus terreus* [36].

Simões et al. [19] reported that a xylanase produced by *M. heterothallica* F.2.1.4 exhibited maximum activity at pH 5.5 and maintained considerable stability under acidic conditions. The similarity between the pH profiles observed in both studies reinforces the robustness of xylanases produced by this species and suggests that *M. heterothallica* consistently produces enzymes adapted to mildly acidic environments.

The peak at pH 5.0 is particularly advantageous, as it coincides with the pH found in several industrial processes, such as hydrolysis of lignocellulosic waste, bioethanol production, and animal feed [37,38]. The broad pH stability exhibited by the enzyme is desirable in industrial processes operating with pH variations, as it reduces the need for constant pH adjustments, contributing to the operational process [39]. In addition, enzymes with broad pH stability tend to perform better in commercial formulations, where shelf stability is also an important criterion.

These characteristics suggest that *M. heterothallica* xylanase is a promising candidate for industrial applications that require resistance to harsh conditions, such as in the textile, food, and biofuel industries. Stability at acidic pH (3.5–4.5) is especially useful in hydrolysis processes of lignocellulosic biomass pretreated with acids, while stability at pH up to 9.5 can be exploited in alkaline processes such as tissue bleaching or paper pulping [40].

**Figure 5:**
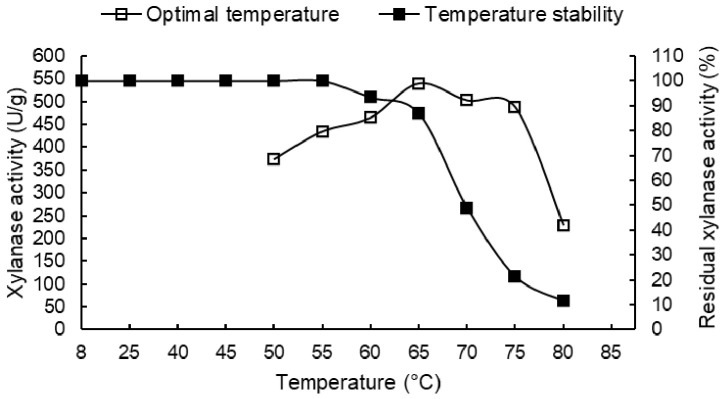
Effect of temperature on the xylanase activity of the fungus *Myceliophthora heterothallica*. -□- optimal temperature; -■- stability at different temperatures, for 1 h.

It was found that the optimal temperature of xylanase was 65 °C and that the enzyme has high thermostability, maintaining more than 85% of its activity for 1 h, up to a temperature of 65 °C (Figure 5).

The xylanase produced by *Myceliophthora heterothallica* showed an optimal activity temperature of 65 °C, in addition to demonstrating high thermostability, maintaining more than 85% of its residual activity after 1 h of incubation at this temperature. These results are indicative of a thermal-cell enzyme with great potential for application in industrial processes operating under high temperatures.

This optimal temperature is in accordance with the profile of xylanases produced by thermophilic fungi, such as *Myceliophthora, Thermomyces, and Thielavia*, which secrete enzymes adapted to environments with high temperatures [37]. This characteristic is desirable because high temperatures in industrial processes can increase the solubility of substrates, reduce the viscosity of mixtures and minimize the risk of microbiological contamination [38]. Similar results were reported by Simões et al. [13,19], who observed optimal activity between 60 and 70 °C for a xylanase produced by M. heterothallica. The optimum temperature of 65 °C observed in the present study confirms the thermophilic nature of the enzymes secreted by this fungus and highlights their potential application in industrial processes requiring elevated temperatures.

The thermal stability of *M. heterothallica xylanase* suggests a robust protein structure, capable of resisting thermal denaturation in aggressive environments. Enzymes with this profile are especially useful in continuous and long-term processes, such as in the production of bioethanol from biomass and in the food industry for juice extraction and clarification [40].

## 4. Conclusions

The combination of sugarcane bagasse, sugarcane straw and wheat bran was the most suitable substrate for xylanase production by *Myceliophthora heterothallica* under solid-state fermentation. Maximum enzyme production reached 533.3 U·g^−1^ dry substrate after four days of cultivation when the substrate was supplemented only with distilled water at pH 5.0, incubated at 40 °C and adjusted to 80% initial moisture.

The xylanase produced exhibited optimum activity at pH 5.0 and 65 °C, maintaining high stability over a broad pH range (3.5–9.5) and retaining more than 85% of its activity after 1 h at 65 °C. These characteristics demonstrate the potential of *M. heterothallica* as a source of thermostable xylanases.

Furthermore, the successful use of sugarcane bagasse, sugarcane straw and wheat bran highlights the feasibility of converting abundant agro-industrial residues into value-added bioproducts, contributing to biomass valorization and supporting future applications in biotechnological and industrial processes.

## Figures and Tables

**Table 1:** Approximate chemical composition of the agro-industrial residues used as substrates for xylanase production.

Substrate	Cellulose (%)	Hemicellulose (%)	Lignin (%)	Protein (%)
Sugarcane bagasse	32–45	20–32	17–32	–
Sugarcane straw	33–40	25–28	21	–
Wheat bran	–	41–60	–	15–18

Source: Adapted from [21,22,23].

## Data Availability

The original contributions presented in this study are included in the article. Further inquiries can be directed to the corresponding author.
